# Comparison of outcomes in ST-segment depression and ST-segment elevation myocardial infarction patients treated with emergency PCI: data from a multicentre registry

**DOI:** 10.5830/CVJA-2012-053

**Published:** 2012-10

**Authors:** Jiri Knot, Fili P Rohac, Robert Petr, Dana Bil Kova, Petr Widimsky, Jan Bělohlavek, Petr Kala, Richard Rokyta, Josef Stasek, Boyko Kuzmanov, Slavejko Djambazov, Mladen Grigorov, Ota Hli Nomaz

**Affiliations:** Third Faculty of Medicine, Charles University, Prague, Czech Republic; Third Faculty of Medicine, Charles University, Prague, Czech Republic; Third Faculty of Medicine, Charles University, Prague, Czech Republic; Third Faculty of Medicine, Charles University, Prague, Czech Republic; Third Faculty of Medicine, Charles University, Prague, Czech Republic; Third Faculty of Medicine, Charles University, Prague, Czech Republic; Faculty Hospital, Brno, Masaryk University, Czech Republic; Faculty of Medicine, Pilsen, Charles University Prague, Czech Republic; Faculty of Medicine, Hradec Kralove, Charles University Prague, Czech Republic; UniCardio Clinic Pleven, Bulgaria; UniCardio Clinic Pleven, Bulgaria; UniCardio Clinic Pleven, Bulgaria; Faculty Hospital St Anne, Masaryk University, Brno, Czech Republic

**Keywords:** coronary artery disease, acute myocardial infarction, primary PCI

## Abstract

**Background:**

Traditionally, acute myocardial infarction (AMI) has been described as either STEMI (ST-elevation myocardial infarction) or non-STEMI myocardial infarction. This classification is historically related to the use of thrombolytic therapy, which is effective in STEMI. The current era of widespread use of coronary angiography (CAG), usually followed by primary percutaneous coronary intervention (PCI) puts this classification system into question.

**Objectives:**

To compare the outcomes of patients with STEMI and ST-depression myocardial infarction (STDMI) who were treated with emergency PCI.

**Methods:**

This multicentre registry enrolled a total of 6 602 consecutive patients with AMI. Patients were divided into the following subgroups: STEMI (*n* = 3446), STDMI (*n* = 907), left bundle branch block (LBBB) AMI (*n* = 241), right bundle branch block (RBBB) AMI (*n* = 338) and other electrocardiographic (ECG) AMI (*n* = 1670). Baseline and angiographic characteristics were studied, and revascularisation therapies and in-hospital mortality were analysed.

**Results:**

Acute heart failure was present in 29.5% of the STDMI vs 27.4% of the STEMI patients (*p* < 0.001). STDMI patients had more extensive coronary atherosclerosis than patients with STEMI (three-vessel disease: 53.1 vs 30%, *p* < 0.001). The left main coronary artery was an infract-related artery (IRA) in 6.0% of STDMI vs 1.1% of STEMI patients (*p* < 0.001). TIMI flow 0–1 was found in 35.0% of STDMI vs 66.0% of STEMI patients (*p* < 0.001). Primary PCI was performed in 88.1% of STEMI (with a success rate of 90.8%) vs 61.8% of STDMI patients (with a success rate of 94.5%) (*p* = 0.012 for PCI success rates). In-hospital mortality was not significantly different (STDMI 6.3 vs STEMI 5.4%, *p* = 0.330).

**Conclusion:**

These data suggest that similar strategies (emergency CAG with PCI whenever feasible) should be applied to both these types of AMI.

## Abstract

ST-segment elevation (STEMI) and ST-segment depression (STDMI) myocardial infarctions have a common pathogenesis – a vulnerable plaque ruptures, followed by luminal thrombus formation.^1^-^4^ Thrombosis may lead to rapid changes in the severity of coronary artery stenosis, which may cause subtotal or total vessel occlusion. The thrombus may completely occlude the major epicardial coronary artery in cases of STEMI,^5^ or cause partial or intermittent vessel occlusion in cases of non-ST-elevation myocardial infarction (NSTEMI).^6^

This traditional classification of patients with acute myocardial infarction (AMI), based on baseline electrocardiographic (ECG) recordings, has practical implications for guidelines and in clinical practice especially, as it refers to the use of reperfusion therapy. The separation of STEMI from other types of acute myocardial infarction has its historical roots in the thrombolytic era.

The current widespread use of primary percutaneous coronary intervention (pPCI) makes use of modified reperfusion treatment for myocardial infarction patients. Recently published randomised trials and meta-analyses,^7^-^12^ as well as the guidelines of the European Society of Cardiology (ESC) for myocardial infarction in patients presenting with persistent ST-segment elevation,^13^ indicate that pPCI is the preferred reperfusion strategy in AMI patients when performed by an experienced team as soon as possible after first medical contact. The pPCI reperfusion modality remains superior to immediate thrombolysis, even if transfer to an angioplasty centre is necessary.

Similarly, an early invasive strategy with early coronary angiography and revascularisation has become the preferred approach for patients with NSTEMI.^14^-^17^ Additionally, the ESC guidelines for the diagnosis and treatment of non-ST-segment elevation acute coronary syndromes (ACI) appropriately recognises AMI with ongoing or recurrent chest pain and ST-segment depression as the highest risk subgroup and is an indication for emergency coronary angiography, followed by revascularisation, when appropriate.^18^ From the sub-analysis of two previously published trials^19^,^20^ and a meta-analysis,^21^ it has been shown that the greatest benefit of early invasive treatment was found in patients with elevated cardiac enzymes and ST-segment changes, i.e. in patients with STDMI.

The aim of this study was to analyse a large group of AMI patients presenting with different ECG records and to assess the similarities and differences between baseline and angiographic characteristics, to assess in-hospital management and mortality, and to test the hypothesis that an emergency PCI strategy should be used in both ST-segment elevation MI as well as in ST-segment depression MI.

## Methods

This retrospective, multicentre, observational registry included a total of 6 602 consecutive patients admitted to five participating centres (four in the Czech Republic and one in Bulgaria; all university-type hospitals with catheterisation facilities) for an acute myocardial infarction during a three-year recruitment period (except for the centre in Bulgaria, where the recruitment period was only one year). All participating hospitals followed the guidelines of the Czech Society of Cardiology.

All patients underwent emergency coronary angiography (CAG). Patients with STEMI, new left bundle branch block (LBBB) or right bundle branch block (RBBB) and STDMI with ongoing chest pain underwent CAG immediately after hospital arrival. In all remaining cases, the procedure was performed within 24 hours of onset of AMI symptoms. Subjects had to be 18 years or older.

Based on admission ECG records, patients were divided into one of five subgroups: ST-elevation AMI (*n* = 3446; 52.2%), ST-depression AMI (*n* = 907; 13.7%), LBBB AMI (*n* = 241; 3.7%), RBBB AMI (*n* = 338; 5.1%), other baseline ECG AMI (*n* = 1670; 25.3%). STEMI was defined as new ST-elevation at the J-point in two contiguous leads with cut-off points of ≥ 0.2 mV in men or ≥ 0.15 mV in women in leads V2–3 and/or ≥ 0.1 mV in other leads. STDMI was defined as a new horizontal or down-sloping ST depression ≥ 0.05 mV in two contiguous leads or transient ST-segment elevations. The other ECG group represented all remaining ECG patterns excluding STEMI, STDMI, LBBB and RBBB.

Patients entered into the registry were admitted for an acute myocardial infarction using only the ESC/ACC myocardial infarction redefinition.^22^ Symptoms consistent with ischaemia, new ECG changes and a typical rise and fall of cardiac enzymes levels (troponin I and/or T and/or creatine phosphokinase-MB) were mandatory for inclusion. Moreover, the diagnosis of MI had to be confirmed at the time of discharge from hospital.

Baseline characteristics, such as age, gender, diabetes mellitus, history of previous myocardial infarction, Killip class on admission and ECG pattern (including information regarding any bundle branch blocks – old, new or of unknown origin) were analysed. Coronary angiographic (or autopsy) data were analysed to estimate the number of diseased major coronary arteries, to identify the infarct-related artery (IRA), and assess thrombolysis in myocardial infarction (TIMI) flow in the infarct-related artery before and after PCI (whenever PCI was performed).

To identify the ejection fraction, pre-discharge echocardiographic examinations were performed. Revascularisation strategies used during the index hospital stay were studied. Patients were followed until transfer to a referral hospital or hospital discharge/death. Death was defined as all-cause mortality during hospitalisation. The in-hospital mortality was also analysed.

## Statistical analysis

Patients with STEMI and STDMI were compared based on demographics, medical history and risk factors, infarct-related artery and segment, initial and post-procedural TIMI flow, reperfusion success and in-hospital mortality. Statistical comparisons between subgroups were performed using Chi-square and Fisher’s exact tests for categorical variables; data are expressed in percentages.

Continuous variables are presented as means ± standard deviations and were compared using the two-sample Student’s *t*-test. For ordinary variables, the Mann–Whitney test was applied. All tests were two-tailed, and a *p*-value < 0.05 was considered statistically significant.

A logistic regression model was used to adjust the differences in mortality for covariate effects. The following factors and covariates were used in the model: age, gender, previous diabetes and myocardial infarction, Killip class > I on admission, and pre-discharge ejection fraction.

## Results

During the study period, a total of 6 602 patients were enrolled in the registry from five participating centres. There were 3 446 patients with STEMI and 907 with STDMI. Patients presenting with STEMI were younger than those with STDMI. The mean age in the STEMI group was 64.5 years and in the STDMI group 69.5 years (*p* < 0.001). There were more patients under 75 years in the group with STEMI than in the STDMI group (74.5 vs 63.6%, *p* < 0.001).

Compared to STEMI patients, STDMI patients were more likely to have a history of a previous MI (STDMI 29.3% vs STEMI 13.8%, *p* < 0.001) and diabetes mellitus (36.8 vs 24.1%, *p* < 0.001). The gender distribution was equal between the STEMI and STDMI groups (females 31.3 vs males 34.6%, *p* = 0.055). Patients in the STEMI group were more likely to be in cardiogenic shock on admission. Killip class IV on admission was present in 6.7% of STEMI patients compared to 4.4% in STDMI patients (*p* < 0.001). Acute heart failure defined as Killip class > 1 on admission (pulmonary rales or third heart sound and pulmonary oedema) was present in 29.5% of STDMI vs 27.4% of STEMI patients (*p* < 0.001) (Table 1).

**Table 1.** Baseline Characteristics In STEMI And STDMI Patients

	*STEMI*	*STDMI*	p*-value*
Age in years ± SD	64.5 ± 12.4	69.5 ± 10.7	< 0.001
Age < 75 years (%)	74.5	63.6	< 0.001
Females (%)	31.3	34.6	0.055
Previous myocardial infarction (%)	13.8	29.3	< 0.001
Diabetes mellitus (%)	24.1	36.8	< 0.001
Killip class > I (%)	27.4	29.5	< 0.001
Killip class IV (%)	6.7	4.4	< 0.001

STEMI: ST-elevation myocardial infarction; STDMI: ST-depression myocardial infarction.

STEMI patients had less-extensive coronary atherosclerosis than STDMI patients. There were more patients with three- or two-vessel disease in the STDMI group than in the STEMI group (73.0 vs 58.2%, *p* < 0.001). Severe left main stenosis was also more often present in STDMI patients compared to STEMI patients (6.0 vs 1.1%, *p* < 0.001). The left circumflex artery was more likely to be the infarct-related artery in STDMI compared to STEMI patients (37.5 vs 14%, *p* < 0.001). Moreover, nearly one-third of all STDMI patients had a TIMI 0 flow before PCI. The infarct-related artery was more often totally occluded in STEMI patients compared to STDMI patients (57.2 vs 27.3%, *p* < 0.001).

**Fig. 1.**
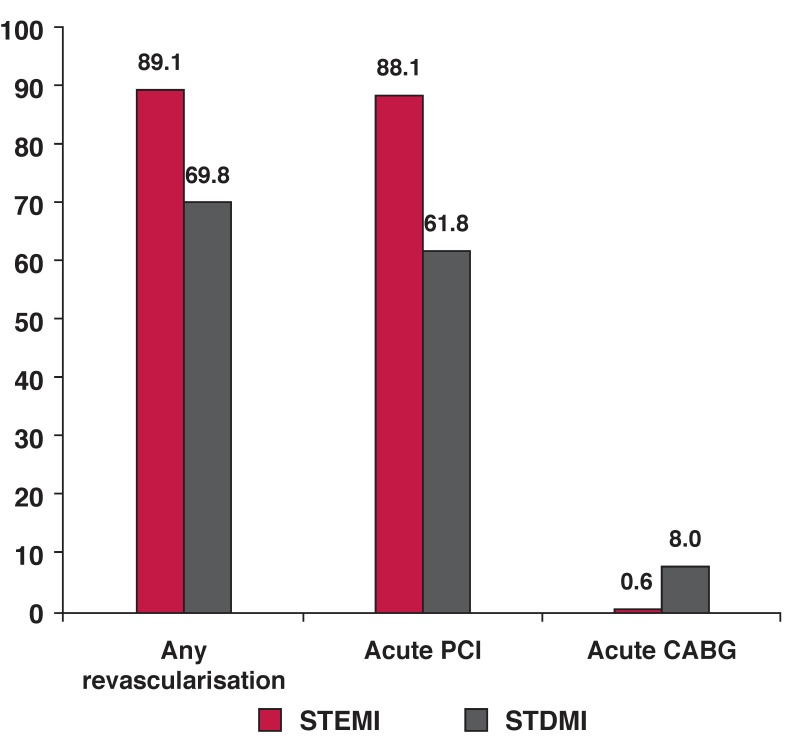
Bar graphs show the type of revascularisation therapy used in ST-segment elevation (STEMI) and ST-segment depression (STDMI) myocardial infarctions. All values are percentages (*p* < 0.001). CABG: coronary artery bypass graft.

Emergency PCI was performed in 88.1% of STEMI patients versus 61.8% of STDMI patients. The success rates were higher in STDMI patients (94.5 vs 90.8%, *p* < 0.012) (Table 2). Rates of acute coronary bypass grafts were significantly higher in patients with STDMI (Fig. 1).

**Table 2.** Angiographic Characteristics And Procedural Outcomes In STEMI And STDMI Patients

	*STEMI*	*STDMI*	p*-value*
Number of involved vessels (%)			
One-vessel disease	37.3	17.2	< 0.001
Two-vessel disease	28.2	19.9	
Three-vessel disease	30.0	53.1	
Infarct-related artery (%)			
Left main	1.1	6.0	< 0.001
Left anterior descending	45.0	31.5	
Left circumflex	14.0	37.5	
Right coronary	39.1	21.2	
CABG	0.8	3.8	
Pre-PCI TIMI flow (%)			
TIMI flow 0	57.2	27.3	< 0.001
TIMI flow 1	8.8	7.7	
TIMI flow 2	18.5	24.5	
TIMI flow 3	15.5	40.6	
Post-PCI TIMI flow (%)			
TIMI flow 3	90.8	94.5	< 0.012
LVEF (%), mean ± SD	46.3 ± 12.0	50.1 ± 13.5	< 0.001

STEMI: ST-elevation myocardial infarction; STDMI: ST-depression myocardial infarction; TIMI: thrombolysis in myocardial infarction flow; CABG: coronary artery bypass graft; PCI: percutaneous coronary intervention; LVEF: left ventricular ejection fraction.

Despite the higher mean ejection fraction, in-hospital mortality was slightly but insignificantly higher in STDMI patients compared to STEMI patients (6.3 vs 5.4%, *p* = 0.330). There was no significant difference regarding in-hospital mortality between STEMI and STDMI patients who were treated using emergency PCI (5.3 vs 6.78%, *p* = 0.274). However, there was a large difference regarding in-hospital mortality between STDMI patients treated using PCI (6.78%) and STDMI patients without revascularisation (13.19%) (*p* = 0.032).

Using logistic regression analysis, the independent risk factor for mortality was patient age (OR 1.03, 95% CI: 1.015–1.049, *p* < 0.001); there was a 1.03-fold increased risk for every additional year of age. Killip class > I on admission was also a strong predictor of mortality (OR 2.54, 95% CI: 1.754–3.685, *p* < 0.001). A lower risk of death was associated with higher ejection fractions (OR 0.982, 95% CI: 0.967–0.997, *p* < 0.024).

Patients presenting with MI and any bundle branch block (left or right bundle branch block ± left anterior/posterior hemiblock) represented the highest risk group, with in-hospital mortality more than double compared to patients who presented with STDMI (risk ratios 2.03, 95% CI: 1.46–2.83, *p* < 0.001) or STEMI (risk ratios 2.36, 95% CI: 1.83–3.04, *p* < 0.001). On the other hand, patients presenting with minor or no ECG abnormalities (without ST-segment shifts and without bundle branch block/s) had a significantly lower risk (acute heart failure was rare and in-hospital mortality was very low). The in-hospital mortality in this group of patients was 2.9% (*p* < 0.001).

**Fig. 2.**
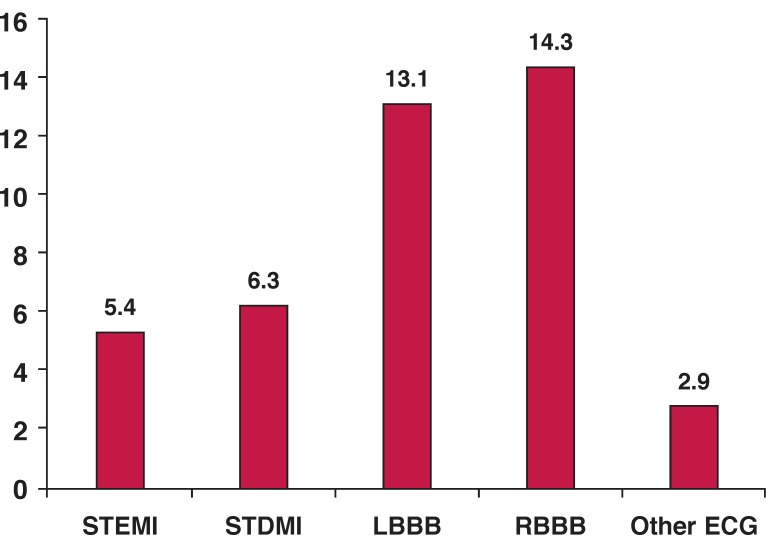
Bar graph demonstrates the in-hospital mortality rates in different ECG groups of acute myocardial infarction patients. All values are percentages (*p* < 0.001). STEMI/STDMI: ST-segment elevation/depression myocardial infarction. LBBB/RBBB: left/right bundle branch block.

Fig. 2 presents a comparison between patients with minor or no ECG changes and each of the other groups (STEMI, STDMI, LBBB, RBBB).

## Discussion

STEMI and STDMI have a common pathogenesis: vulnerable plaque erosion or rupture followed by thrombus formation, resulting in impaired vessel patency. Impaired or no flow in a coronary artery causes ischaemic symptoms and ECG changes. The release of myocardial necrosis markers defines the diagnosis of myocardial infarction.

The current guidelines recommend different reperfusion approaches based on the admission ECG in patients with acute MI. On the other hand, ECG changes can be altered by a bundle branch block, previous MI and other conditions. Also, the infarct-related artery and infarct-related segment can influence the final ECG pattern. For example, acute occlusion of the circumflex artery may have no ST-segment elevation on a 12-lead ECG. Instead, ST-segment depressions are frequently present – this is sometimes called a hidden STEMI.

In our registry the most common IRA in STDMI patients was the circumflex branch. Moreover, nearly one-third of all STDMI patients had a TIMI grade 0 flow before PCI. Infarction in the circumflex artery bed is very often under-diagnosed and these patients undergo coronary angiography very late or not at all. Based on these facts, there is an increasing effort to find real differences or similarities between STEMI and STDMI regarding their risk factors, prognosis, mortality and appropriate revascularisation strategy.

In previously published studies, baseline characteristics of patients with STEMI compared to those without ST-segment elevation were significantly different, and the same was true in this study. Patients with STEMI were younger and had less often had a previous MI and/or diabetes mellitus. Cardiogenic shock was also found to be more common in STEMI patients.

Rosenberger *et al*.^23^ investigated whether risk factors were related differently to ST-elevation and non-ST-elevation ACS. The main finding from this large survey of more than 10 000 patients was that different risk factors were related to different types of ACS. Smoking was related to STEMI patients, whereas obesity and high blood pressure were more common among MI patients without ST-elevation.

Our findings confirm the results of the Opera registry.^24^ The primary objective of the nationwide Opera study was to describe the in-hospital management and cardiovascular outcomes (at one year) of MI patients. The results show that patients suffering from MI with and without ST-elevation had comparable in-hospital (4.6 vs 4.3%) and long-term prognoses (9% in STEMI vs 11.6% in NSTEMI, log-range *p* = 0.09).

Cox *et al*.^25^ showed (in the Comparative early and late outcomes after primary percutaneous coronary intervention in ST-segment elevation and non-ST-segment elevation acute myocardial infarction from the CADILLAC trial) that patients with myocardial infarction without ST-elevation tended to have lower mortality rates than those with STEMI (0.4 vs 2.2%, *p* = 0.06). Similarly, the mortality rates at one year were comparable in STEMI and NSTEMI patients (3.4 vs 4.4%, respectively, *p* = 0.43). In a study by Savonitto *et al*.,^26^ the 30-day mortality rate between STEMI and STDMI was not statistically different (5.1 vs 5.1%, respectively).

Granger *et al*.^27^ attempted to develop a single model to assess the risk for in-hospital mortality of ACS patients. Killip class was the most powerful predictor with a two-fold increased risk of death with each worsening class. Age was associated with nearly the same prognostic significance, with a 1.7-fold increased risk for every 10 years’ increase in age.

The next most important variables were systolic blood pressure, resuscitated cardiac arrest and initial serum creatinine levels. The strongest predictors of one-year mortality in the Opera study were heart failure and age. Moreover, similar predictors were found in STEMI and NSTEMI patients.^24^ The same was true in our registry, with age and heart failure being strong independent in-hospital mortality risk factors.

There is no doubt that timely reperfusion of STEMI patients is critical. The current guidelines of the European Society of Cardiology appropriately recognise acute myocardial infarction with ongoing or recurrent chest pain and ST-segment depressions as the highest-risk subgroup and an indication for emergency coronary angiography, followed by revascularisation when appropriate.

Chan *et al*.^28^ investigated mortality differences and timing of revascularisation of patients undergoing cardiac catheterisation for STEMI and NSTEMI. During the six-year accrual period, a total of 1 974 patients were classified as having STEMI, and 2 413 patients as having NSTEMI. NSTEMI was associated with a higher risk of long-term mortality (unadjusted mortality at one year for STEMI was 9.5 vs 14.3% for NSTEMI). Compared with no or late revascularisation, early revascularisation was associated with a similar reduction in long-term outcomes for both STEMI and NSTEMI (lower adjusted risk of mortality for STEMI and NSTEMI, *p* = 0.22).

The Fragmin and Fast Revascularisation during InStability in Coronary artery disease (FRISC-2) invasive trial showed for the first time a significant event rate (MI, death or both) reduction, favouring the invasive over the non-invasive strategy at six months in the NSTE-ACS population. The greatest benefit of invasive treatment, when evaluated using electrocardiography, was seen in the subset of patients with ST-segment depression MI.^19^ The Treat Angina with Aggrastat and Determine Cost of Therapy with an Invasive or Conservative Strategy (TACTICS-TIMI) trial revealed that the greatest benefits of invasive treatment were achieved in patients presenting with cardiac enzyme elevation and ST-segment changes,^20^ i.e. in STDMI patients. Also, a meta-analysis of seven randomised trials that included a total of 9 212 patients showed that invasive management should be considered for all patients with NSTEMI, and in particular those with ST-segment depression.^21^

In our study, there was no difference related to in-hospital mortality between STEMI and STDMI patients treated by emergency PCI (5.3 vs 6.78%, respectively, *p* = 0.274). There was a significant in-hospital mortality reduction in STDMI patients who were treated using emergency PCI compared to STDMI patients who went without revascularisation. Moreover, the PCI success rate was significantly higher in STDMI compared to STEMI patients (*p* = 0.012). All these factors indicate that emergency CAG and PCI, when appropriate, should be used in all STDMI patients.

Early versus delayed invasive intervention in patients with ACS without ST-segment elevation was studied in the TIMACS trial.^29^ Early intervention did not differ greatly from delayed intervention in preventing the primary outcome, but it did reduce the rate of the composite secondary outcome of death, MI or refractory ischaemia, and was superior to delayed intervention in high-risk patients.

Our study demonstrates the apparent positive development in invasive reperfusion treatment for acute myocardial infarction. Some form of reperfusion therapy was used in 89.1% of STEMI and 69.8% of NSTEMI patients. Of those, emergency PCI was used in 88.1% of STEMI and in 61.8% of NSTEMI patients.

By comparison, in the GRACE study (1999–2000),^30^ the use of pPCI was a relatively rare reperfusion modality in STEMI. Lytic therapy was used in more than 75% of patients who received reperfusion therapy; only 62% of STEMI patients received any form of reperfusion. The in-hospital fatality rates were 7% in STEMI and 6% in NSTEMI patients (*p* = 0.0459). This positive and increasing trend of invasive treatment in AMI patients should be minimally maintained in STEMI cases, and there should be an effort made to increase the number in STDMI patients.

The presence of bundle brunch block(s) (BBBs) is associated with poor outcomes in patients suffering from an AMI. In our MI population, these patients represented the highest risk group, with in-hospital mortality more than double that of STDMI or STEMI patients. Patients with BBBs were older and more frequently had a history of diabetes mellitus. The mean left ventricular ejection fraction was lower compared with AMI patients without BBBs (*p* < 0.001). These findings support the results of Guerrero *et al*.^31^ who sought to evaluate the outcome of patients with AMI and BBBs, who were treated using emergency PCI. The in-hospital mortality was significantly different (LBBB 14.6% vs RBBB 7.4% vs no BBB 2.8%).

Patients presenting with minor or no ECG abnormalities (without ST-segment shifts and without a bundle branch block) had the lowest mortality compared with all other groups (2.9%, *p* < 0.001). Additionally, heart failure was rare (Killip class I on admission was seen in 84.5% of all patients in this group).

## Limitations

This study was based on the data from a registry that was retrospectively analysed. The very short follow-up period was a limitation. Our results did not evaluate long-term outcomes. No data were collected regarding previous or in-hospital drug treatment. Post-discharge treatment (secondary prevention) was also not studied.

## Conclusions

The results of our study demonstrate that ST-depression AMI may represent an emergency similar to ST-elevation AMI. Therefore it would be accompanied by the same need for emergency coronary angiography and PCI when appropriate. STDMI patients in our study had comparable in-hospital mortality and were much closer, relative to treatment strategies and outcomes, to STEMI patients than to AMI patients without ST-segment shifts. Therefore, in the ‘post-thrombolytic’ era, emergency CAG and PCI, when appropriate, should be considered for high-risk patients with STDMI.
